# Transcriptome wide identification and characterization of starch branching enzyme in finger millet

**DOI:** 10.6026/97320630013179

**Published:** 2017-06-30

**Authors:** Rajhans Tyagi, Apoorv Tiwari, Vijay Kumar Garg, Sanjay Gupta

**Affiliations:** 1 Uttarakhand Technical University, Dehradun, Uttarakhand, 248007, India; 2 Sam Higginbottom University of Agriculture, Technology And Sciences (SHUATS), Allahabad, 211007, India; 3 Dept of Molecular Biology and Genetic Engineering, G.B. Pant University of Agriculture and Technology, Pantnagar, Uttarakhand, 263145, India

**Keywords:** Finger millet, CDS, SBE, Domain, PMDB, NCBI

## Abstract

Starch-branching enzymes (SBEs) are one of the four major enzyme classes involved in starch biosynthesis in plants and play an important role in determining the structure and physical properties of starch granules. Multiple SBEs are involved in starch biosynthesis in plants. Finger millet is calcium rich important serial crop belongs to grass family and the transcriptome data of developing spikes is available on NCBI. In this study it was try to find out the gene sequence of starch branching enzyme and annotate the sequence and submit the sequence for further use. Rice SBE sequence was taken as reference and for characterization of the sequence different in silico tools were used. Four domains were found in the finger millet Starch branching enzyme like alpha amylase catalytic domain from 925 to2172 with E value 0, N-terminal Early set domain from 634 to 915 with E value 1.62 e-42, Alpha amylase, C-terminal all-beta domain from 2224 to 2511 with E value 5.80e-24 and 1,4-alpha-glucan-branching enzyme from 421 to 2517 with E value 0. Major binding interactions with the GLC (alpha-d-glucose), CA (calcium ion), GOL (glycerol), TRS (2-amino-2-hydroxymethylpropane- 1, 3-diol), MG (magnesium ion) and FLC (citrate anion) are fond with different residues. It was found in the phylogenetic study of the finger millet SBE with the 6 species of grass family that two clusters were form A and B. In cluster A, finger millet showed closeness with Oryzasativa and Setariaitalica, Sorghum bicolour and Zea mays while cluster B was formed with Triticumaestivum and Brachypodium distachyon. The nucleotide sequence of Finger millet SBE was submitted to NCBI with the accession no KY648913 and protein structure of SBE of finger millet was also submitted in PMDB with the PMDB id - PM0080938. This research presents a comparative overview of Finger millet SBE and includes their properties, structural and functional characteristics, and recent developments on their post-translational regulation.

## Background

A cereal is a grass, a member of the monocot family poaceae also known as gramineae, which usually have long, thin stalks, such as wheat, rice, maize, sorghum, millet, barley and rye, whose starchy grains are used as food. The term cereal is not limited to these grains, but, also refers to foodstuff prepared from the starchy grains of cereal like flours, breads and pasta [9]. All cereals are annual plants and consequently, one planting yields one harvest. The demands on climate, however, are different. Warm-season cereals (corn, rice, sorghum, millet) are grown in tropical lowlands throughout the year and in temperate climates during the frost- free season. Rice is mainly grown in flooded fields, and sorghum and millet are adapted to arid conditions. Cool-season cereals (wheat, rye, barley, and oats) grow best in a moderate climate. Finger millet is considered to be a boon for diabetes patients and obese people, as the digestion of finger Millet takes place at a slow pace and hence, glucose is released slowly into the blood [2]. Starch-branching enzymes (SBEs) are one of the four major enzyme classes involved in starch biosynthesis in plants [4]. Starch is the important molecule for plant development and reproduction. Re-structuring of starch granules in-planta can affect plant metabolism and by the research it was proved that the starch branching enzymes play an important role in the Re-structuring of starch granules [10]. Starch can define as a complex branched glucose polymer and the branch molecular weight distribution powerup the nutritionally important properties such as digestion rate, etc [6]. By different studies it was also found that different branching enzyme isoforms contribute separately to the synthesis and final structure of amylopectin [12]. One research describe that the comprehensive in vitro studie revealed different enzymatic characteristics of the BE isozymes and observed the important roles of BE isozymes in amylopectin biosynthesis in the endosperm [8]. In rice and other related plants of grass family like Oryzasativa and Setariaitalica, Sorghum bicolour and Zea mays while cluster B was formed with Triticumaestivum and Brachypodiumdistachyon the sequence of starch branching enzyme is known but in finger millet the nucleotide sequence of the Starch branching enzyme is not yet known. Availability of the transcriptome data of developing spikes of finger millet provide an opportunity to predict the gene sequence of important traits of nutritional point view. Hence an effort is made for the prediction of an important enzyme i.e. starch branching enzyme in the finger millet and submit the genomic as well as proteomic information for further scientific and research interventions.

## Methodology

### Sequence Retrieval

The sequence of Starch branching enzyme of finger millet was retrieved by the blast analysis of homologous sequence of rice obtained from NCBI. Local BLAST [1] was used to retrieve the sequence of Starch branching enzyme of finger millet from the transcriptome data of finger millet of developing spikes.

### ORF Prediction

The open reading frame analysis of starch branching enzyme was completed using ORF prediction tool at NCBI (https://www.ncbi. nlm.nih.gov/orffinder) [13]. The minimal ORF length in nucleotide was set to 75 and ATG codon was used as start codon for the ORF translation.

### Domain Analysis

After prediction of ORF we had gone prediction of domain in the coding region of starch branching enzyme (homologous to rice). CD-Search tool of NCBI [7] was used for the prediction of domain in the cds sequence of finger millet. The search database CDD V3.15-48963 PSSMs was used at the expect value 0.010000(https://www.ncbi.nlm.nih.gov/Structure/cdd/wrpsb. cgi).

### Phylogenetic analysis

Phylogenetic analysis of the starch branching enzyme of finger millet was done by the two ways. In the first way the phylogenetic study was done on the basis of blast result of NCBI, total hit sequences were taken for the multiple sequence alignment and then gone for phylogenetic analysis by the neighbour joining method using MEGA6 tool [13]. In other hand the phylogenetic was done only the species of grass family including Setariaitalica, Triticumaestivum, Sorghum bicolour, Brachypodiumdistachyon, Zea mays, Oryzasativa and finger millet.

### Structure Analysis

The protein sequence of the coding sequence of starch branching was used for the tertiary structure prediction. RaptorX structure prediction server [5] was used for prediction of tertiary structure of the finger millet starch branching enzyme.(http://raptorx.uchicago.edu/StructurePrediction/predi ct/).

### Model-assisted Protein Binding Site Prediction

RaptorX server was used for prediction of binding site in the protein structure of the starch synthase enzyme of finger millet. (http://raptorx.uchicago.edu/BindingSite/) [13].

### Sequence and structure submission

NCBI Bankit server was used for submission of nucleotide sequence as protein sequence of starch branching enzyme of finger millet. While in other hand for submission of protein structure of the starch branching enzyme the PMID Protein Model DataBase, which collects three dimensional protein models obtained by structure prediction methods.

## Results and Discussion

### ORF Prediction

**Figure 1:**
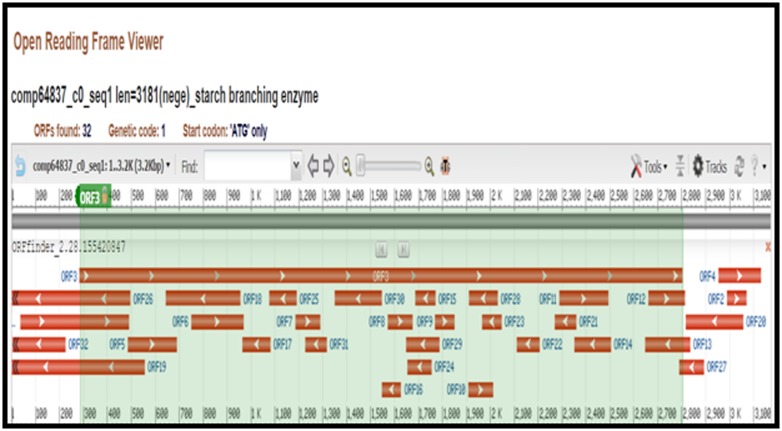
ORF prediction in the finger millet Starch branching enzyme

The ORF prediction tool in the SBE of finger millet found total 32 ORFs and ORF 3 was selected because it is the longest orf with the 2530 nucleotide long and 839 amino acid residues in protein sequence (Figure 1).

### Domain Analysis

**Figure 2:**
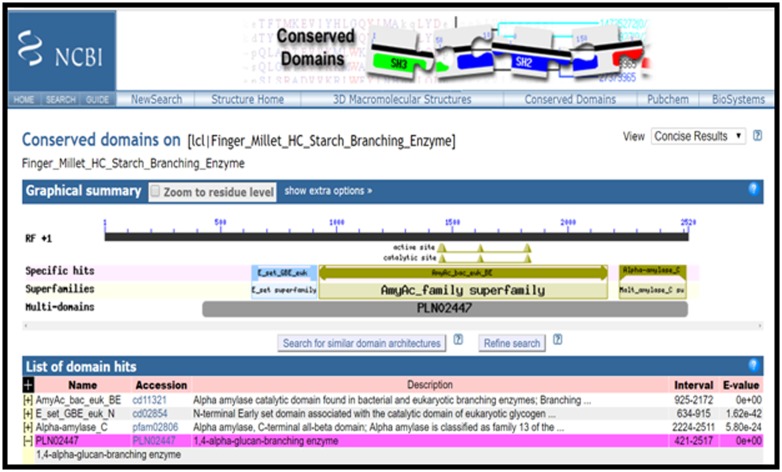
Domain analysis of finger millet starch branching enzyme.

Total four domains were found in the coding sequence of starch branching enzyme of finger millet. AmyAc_baceuk_BE (Alpha amylase catalytic domain) from 925 to2172 with E value 0, E_set_GBE_EUK_N (N-terminal Early set domain) from 634 to 915 with E value 1.62e-42, Alpha-amylase_C (Alpha amylase, Cterminal all-beta domain) from 2224 to 2511 with E value 5.80e-24 and PLN02447 (1,4-alpha-glucan-branching enzyme) from 421 to 2517 with E value 0. (Figure 2)

Alpha amylase catalytic domain [which have specific hit (evalue = 0e+00)] found in eukaryotic branching enzymes which catalyze the formation of alpha-1, 6 branch points in either glycogen or starch by cleavage of the alpha-1, 4 glucosidic linkage yielding a non-reducing end oligosaccharide chain, and subsequent attachment to the alpha-1, 6 position.

N-terminal Early set domain associated with the catalytic domain of eukaryotic glycogen branching enzyme (also called 1,4 alpha glucan branching enzyme); This subfamily is composed of predominantly eukaryotic 1,4 alpha glucan branching enzymes, also called glycogen branching enzymes or starch binding enzymes in plants. E or "early" set domains are associated with the catalytic domain of the 1,4 alpha glucan branching enzymes at the N-terminal end. These enzymes catalyze the formation of alpha-1, 6 branch points in either glycogen or starch by cleavage of the alpha-1,4 glucosidic linkage, yielding a non-reducing end oligosaccharide chain, as well as the subsequent attachment of short glucosyl chains to the alpha-1,6 position. Starch is composed of two types of glucan polymer: amylose and amylopectin.

Alpha amylase, C-terminal all-beta domain; Alpha amylase is classified as family 13 of the glycosyl hydrolases. The structure is an 8 stranded alpha/beta barrel containing the active site, interrupted by a ∼70 a.a. calcium-binding domain protruding between beta strand 3 and alpha helix 3, and a carboxyl-terminal Greek key beta-barrel domain.

### Phylogenetic analysis

**Figure 3:**
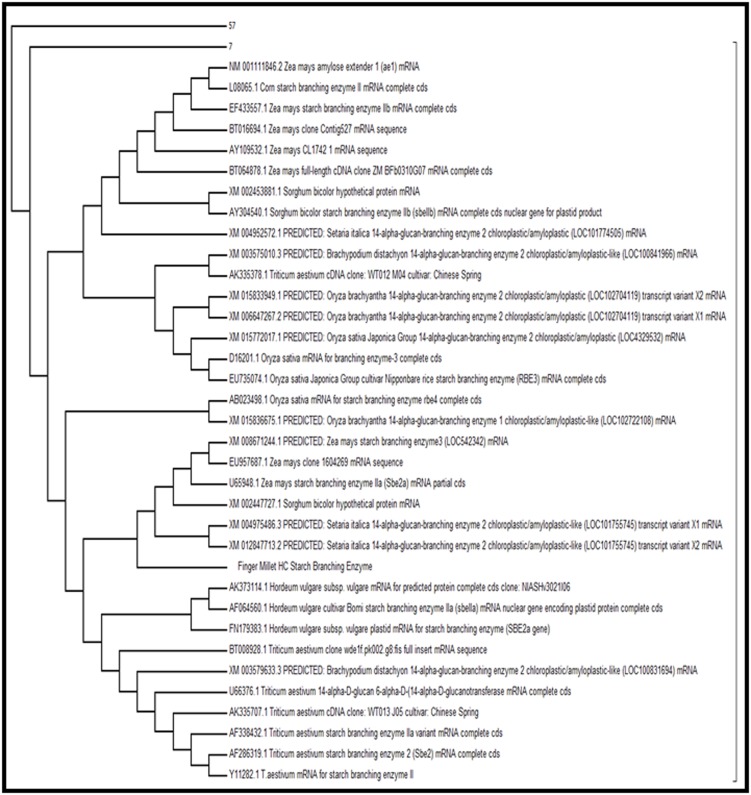
Phylogenetic analysis of finger millet SBE with the total blast hit sequences.

**Figure 4:**
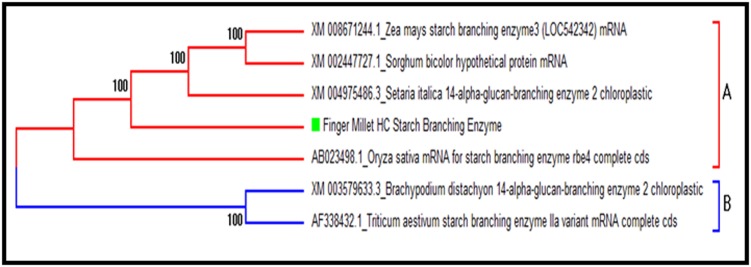
Phylogenetic analysis within grass family.

The evolutionary history was inferred using the Neighbor- Joining method. The percentage of replicate trees in which the associated taxa clustered together in the bootstrap test (1000 replicates). The tree is drawn to scale, with branch lengths in the same units as those of the evolutionary distances used to infer the phylogenetic tree. The evolutionary distances were computed using the Maximum Composite Likelihood method and are in the units of the number of base substitutions per site. The analysis involved 99 nucleotide sequences, codon positions included were 1st+2nd+3rd+Noncoding. All ambiguous positions were removed for each sequence pair. There were a total of 2544 positions in the final dataset. Evolutionary analyses were conducted in MEGA6. (Figure 3) when we use the 99 nucleotide sequences then we found that different clusters were formed according to their sequence level similarity and a big tree was generated but we collapse the branches which were far away from the finger millet as shown in the below figure in one cluster 57 and in other 7 sequences were collapsed. To understand the clear phylogenetic relationship with the species of grass family, another tree was constructed as shown in Figure 4.

It was found in the phylogenetic study of the finger millet SBE with the 6 species of grass family that two clusters were form A and B. In cluster A, finger millet showed closeness with Oryzasativa and Setariaitalica, Sorghum bicolour and Zea mays while cluster B was formed with Triticumaestivum and Brachypodiumdistachyon (Figure 3 and 4).

### Protein Structure Prediction

**Figure 5:**
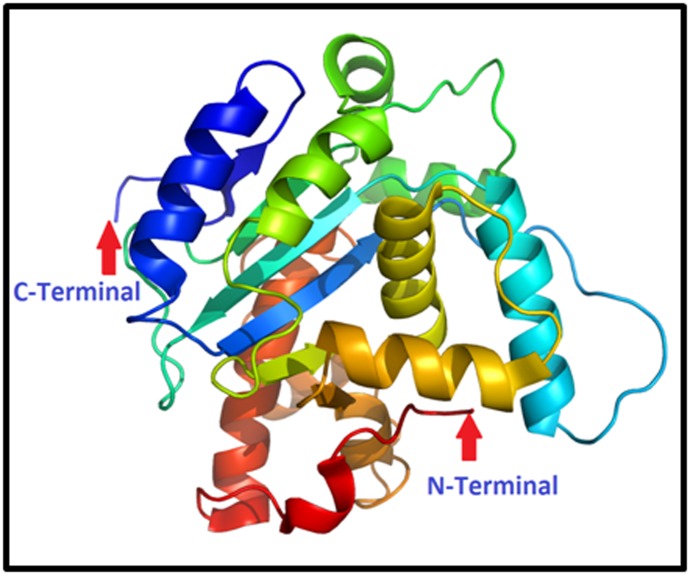
3D Structure of finger millet Starch Branching Enzyme

The input predicted as 2 domain(s) and best template for finger millet SBE was found is 3amlA with p-value 1.11e-27. Overall uGDT (GDT) is 609 (72), 756(90%) residues are modelled, 152(18%) positions predicted as disordered. If we talk about the secondary structure of the enzyme then 19%H, 15%E, and 65%C were found. The solvent access were found in the enzyme sequence is 27%E, 36%M, and 36%B. The protein 3D structure was submitted in PMDB database as in the form of pdb. The PMDB id of the protein sequence is PM0080938 (Figure 5).

### Model-assisted Protein Binding Site Prediction

The RaperX server in the finger millet SBE but in this result predicted total 10 binding sites we select one for each ligand. Major binding interactions with the GLC (alpha-d-glucose), CA (calcium ion), GOL (glycerol), TRS (2-amino-2-hydroxymethylpropane- 1, 3-diol), MG (magnesium ion) and FLC (citrate anion). The corresponding residues with the position are also given in detail in the Table 1.

## Conclusion

Finger millet is a nutritionally rich cereal crop and grown in different regions of worlds and even in adverse weather conditions. It has the abundant amount of calcium comparatively all the cereal crops. The transcriptome data of finger millet is also available on NCBI but the genome of this important crop is not yet available. Different pathways are involved in the starch biosynthesis with the presence of different enzymes. In finger millet Starch branching enzyme nucleotide and protein sequence is not yet known but in this research it was an attempt to predict the genomic and proteomic information of SBE of finger millet, which plays an important role in the synthesis of carbohydrates. The predicted nucleotide sequence and protein structure is available in NCBI and PMDB respectively for further scientific use. This is a first novel attempt for the characterization of enzyme involved in starch biosynthesis in finger millet.

## Figures and Tables

**Table 1:** Model-assisted Protein Binding Site Prediction in the protein sequence of Finger millet starch branching enzyme.

Pocket	Multiplicity	Ligand	Binding residues
1	46	GLC (ALPHA-D-GLUCOSE)	Y374 S377 F378 Y380 H381 W451 D452 Q610 A611 L612 F679
2	20	CA (CALCIUM ION)	I298 Y300 E371 H372 S373 S389
3	18	GOL (GLYCEROL)	C550 P552 G559 F560 D561 K600
4	15	TRS (2-AMINO-2-HYDROXYMETHYL-PROPANE-1,3-DIOL)	L575 S579 D580 R643 L647 W768 R829
5	14	MG (MAGNESIUM ION)	H747 Y775 R776
6	13	FLC (CITRATE ANION)	K577 L612 V613 G614 D615 W621
